# Special Hospitals: IV. Lying-In and Women

**Published:** 1893-09-23

**Authors:** 


### The Institutional Workshop.

### HOSPITAL ADMINISTRATION.

### IV.—SPECIAL HOSPITALS: LYING-IN AND WOMEN.

Cost of Management.*

It is not easy to deal with the lying-in hospitals. They occupy a place entirely different from the general hospitals, owing to the character of the work done, and the nature of the cases. As a rule the patients reside but a very short time in the wards; their general health is good from first to last, and the expenditure upon the in-patients can therefore be reduced to a relatively small sum. The secretary of a lying-in hospital has, however, to raise the necessary income by the same methods as other hospital authorities, and so the cost of management to maintenance must be treated on the same lines. We are doubtful, however, if lying-in hospitals excite at the present day anything like equal sympathy with the general and children's hospitals, and bearing in mind the comparatively small size of these institutions, it would be reasonable to expect that the percentage of cost of administration to that of maintenance would average upwards of 15 per cent., though it should not exceed 20 per cent. Bearing this in mind, let us see how the figures work out.

Metropolitan Lying-In Hospitals.

| Name of Hospital. | Beds for Occupation. | Average Number Occupied. | Total In-patients Admitted. | Total Out-patients Admitted. | Percentage of cost of Administration to that of Maintenance. | Percentage of Administration to Total Expenditure. |
|---|---|---|---|---|---|---|
| Queen Charlotte's ... ... | 58 | 38 | 972 | 1,133 | 11·509 | 10·321 |
| City of London ... ... | 36 | 24 | 487 | 1,536 | 10·077 | 9·188 |
| General Lying-In ... ... | 24 | 20 | 490 | 1,291 | 10·672 | 9·643 |
| Clapham Maternity... ... | 21 | 9 | 206 | 370 | 8·294 | 7·659 |
| British Lying-In ... ... | 16 | 7 | 153 | 435 | 8·450 | 7·792 |
| East-End Mothers' Home ... | 12 | 6 | 140 | 201 | 26·568 | 20·991 |

The most noticeable feature in the above table, as compared with that of last year, is the enormous improvement in the management of the East-end Mothers' Home. The percentage of cost of administration to maintenance has been reduced from 43·8 per cent. to 26·568 per cent., the percentage of cost of administration to total ordinary expenditure being less than 1 per cent. over the maximum we have fixed as being permissible in certain cases. The in-patients, too, have increased from 86 to 140, and out-patients from 28 to 101. We congratulate the new management upon this remarkable reduction, which reflects the greatest credit upon the institution, and should secure for it a much larger measure of public interest and support. The managers of the other lying-in hospitals have evidently been conscientiously and successfully endeavouring to reduce expenditure, as the percentage of cost of administration to maintenance, as compared with 1891, has been reduced in 1892, at the British Lying-in Hospital from 10·931 to 8·450 per cent., and at the Clapham Maternity from 9·885 to 8·294—facts which entitle the managers to warm commendation. There has been a slight increase in the cost of administration at the City of London and General Lying-in of about 1¼ and 1 per cent. respectively. These increases are due no doubt to the deserved recognition of the able services rendered by some officials, Mr. R. A. Owthwaite being, for instance, one of the most zealous and able of special hospital secretaries. The average cost of administration of all the hospitals proper is again less than half the maximum sum—which we put down at 20 per cent.—which it is reasonable to allow for the expenses of management. These lying-in hospitals do an enormous amount of good, and for the relief they afford, and as centres for the instruction of nurses, they deserve a larger measure of public support and recognition than they have so far received. It will be noticed that the difference between the percentage of cost of administration to maintenance and to total ordinary expenditure respectively is no larger in this class of hospital than in that of others with which we have dealt in previous articles.

Diseases Peculiar to Women.

There are six hospitals in London which confine their attention to the diseases peculiar to women, but of these two—the Grosvenor and the Royal—admit a certain number of children also. This is a time when women's rights are much to the fore, and there has never been an age more in sympathy with the best interests of women than that in which we live. These considerations lead us to conclude that 15 per cent. is a fair average sum to fix as the maximum which may be spent upon management. The following is a list of the metropolitan women's hospitals:—

Metropolitan Hospitals for Women.

| Name of Hospital. | Beds for Occupation. | Average Number Occupied. | Total In-patients Admitted. | Total Out-patients Admitted. | Percentage of cost of Administration to that of Maintenance. | Percentage of Administration to Total Expenditure. |
|---|---|---|---|---|---|---|
| Hospital for Women, Soho Square ... ... | 64 | 44 | 530 | 5,153 | 17·247 | 14·711 |
| Chelsea Hospital ... ... ... ... | 60 | 40 | 518 | 3,493 | 24·612 | 20·000 |
| Samaritan Free ... ... ... ... | 53 | 45 | 562 | 8,339 | 14·576 | 12·722 |
| Royal Hospital, Lambeth ... ... ... | 51 | 50 | 576 | 7,193 | 20·094 | 16·732 |
| New Hospital ... ... ... ... ... | 42 | 35 | 500 | 9,484 | 16·247 | 13·976 |
| Grosvenor ... ... ... ... ... ... | 18 | 12 | 130 | 1,436 | 11·746 | 10·511 |

* Previous articles on this subject have appeared in The Hospital: Page 333, for August 19th; page 349, for August 26th; and page 381, for September 9th.

We are glad to see that the anomalous position occupied by this class of hospital last year, when of the six institutions those which contain the greatest number of beds for women showed the greatest expenditure upon management, has been altered by the care of the authorities of the Hospital for Women, Soho Square. This institution has reduced the percentage of cost of administration to maintenance in 1892, as compared with 1881, by no less than 3 per cent.; that is to say, the cost has been reduced from 20·248 to 17.247 per cent. The percentage of cost of administration to total ordinary expenditure at the Hospital for Women is only 14·711 per cent., so that it is entitled to take its place as one of the best managed special hospitals.

There has been a further reduction at the Samaritan Free Hospital, where the percentage of cost of administration to maintenance has fallen from 14·723 in 1891, a comparatively low percentage, to 14·576 in 1892. The percentage of cost of administration to total ordinary expenditure at the Samaritan Free Hospital is only 13¾ per cent., a fact which proves Mr. George Scudamore to be one of the ablest of hospital economists. The Samaritan Free Hospital is probably the best known of all women's hospitals, because of the famous work done within its walls by Sir Spencer Wells. The institution has had many and great difficulties to contend with, and, as we said on a former occasion, it is not too much to say that the results shown by the figures in the table bear eloquent testimony, not only to the efficiency of Mr. Scudamore, but to the care which he exercises in the administration of one of the best managed of special hospitals.

The New Hospital for Women is entirely managed by women, and although its percentage of cost of administration to maintenance has increased by rather more than 1 per cent. in 1892, as compared with 1891, the percentage of cost of administration to total ordinary expenditure is still only 13·976 per cent. We congratulate the ladies who take so active an interest in the New Hospital upon this satisfactory result.

The Grosvenor Hospital, a relatively small institution, with only 18 beds, of which 12 are constantly occupied spends less than 12 per cent. upon its management, the percentage of cost of administration to total ordinary expenditure being but 10·511 per cent. We are inclined to think that the committee of the Grosvenor Hospital would be well advised to increase the emoluments of the secretariat, and to spend a little more in raising money so as to enable them to keep a larger number of beds constantly occupied.

On the other hand, we regret to see that the Chelsea Hospital for Women, which expended the large sum, of 23·119 in 1891 upon administration as compared with maintenance, has increased this outlay to 24·612 in 1892. The percentage of cost of administration to total ordinary expenditure is 20 per cent., which is some 6 per cent. above the average expenditure under this head of all the remaining Metropolitan hospitals for Women. We hope the authorities will give some explanation of the causes which have produced this extraordinary result, for without it many people may be induced to pause before further subscribing to its funds.

The Royal Hospital for Women and Children, Lambeth, increased its percentage of cost of administration to maintenance from 19·225 in 1891 to 20·094 in 1892. Despite this the percentage of cost of administration to total ordinary expenditure was only 16⅔ per cent., and it should not be difficult for the authorities to reduce this latter item by 1¼ per cent., so as to bring it into the category of the best managed institutions in this country.

It affords us pleasure to commend the Samaritan Free Hospital, the New Hospital for Women, and the Hospital for Women, Soho Square, to the favourable consideration of the wealthy, as being on the whole the most efficiently conducted of women's hospitals. These institutions contain every modern appliance, and the internal administration leaves little or nothing to be desired, although, as we have shown, the percentage of management is relatively so small. We have no doubt that the committees of these three hospitals will be gratified to find that they compare most favourably with other women's hospitals throughout the world, and that they will take an opportunity of expressing their appreciation of the devoted service of those officers connected with them who have by continuous effort placed them in such an enviable position.

